# *Trichoderma asperellum* T76-14 Released Volatile Organic Compounds against Postharvest Fruit Rot in Muskmelons (*Cucumis melo*) Caused by *Fusarium incarnatum*

**DOI:** 10.3390/jof7010046

**Published:** 2021-01-12

**Authors:** Warin Intana, Suchawadee Kheawleng, Anurag Sunpapao

**Affiliations:** 1 School of Agricultural Technology and Food Industry, Walailak University, Tha Sala, Nakhon Si Thammarat 80161, Thailand; iwarin@wu.ac.th; 2 Graduate School, Prince of Songkla University, Hatyai 90112, Thailand; 5910610056@psu.ac.th; 3 Agricultural Innovation and Management Division, Faculty of Natural Resources, Prince of Songkla University, Hatyai 90112, Thailand

**Keywords:** muskmelon, biocontrol agent, volatile compounds, *Trichoderma*

## Abstract

Postharvest fruit rot caused by *Fusarium incarnatum* is a destructive postharvest disease of muskmelon (*Cucumis melo*). Biocontrol by antagonistic microorganisms is considered an alternative to synthetic fungicide application. The aim of this study was to investigate the mechanisms of action involved in the biocontrol of postharvest fruit rot in muskmelons by *Trichoderma* species. Seven *Trichoderma* spp. isolates were selected for in vitro testing against *F. incarnatum* in potato dextrose agar (PDA) by dual culture assay. In other relevant works, *Trichoderma asperellum* T76-14 showed a significantly higher percentage of inhibition (81%) than other isolates. Through the sealed plate method, volatile organic compounds (VOCs) emitted from *T*. *asperellum* T76-14 proved effective at inhibiting the fungal growth of *F. incarnatum* by 62.5%. Solid-phase microextraction GC/MS analysis revealed several VOCs emitted from *T*. *asperellum* T76-14, whereas the dominant compound was tentatively identified as phenylethyl alcohol (PEA). We have tested commercial volatile (PEA) against in vitro growth of *F. incarnatum*; the result showed PEA at a concentration of 1.5 mg mL^−1^ suppressed fungal growth with 56% inhibition. Both VOCs and PEA caused abnormal changes in the fungal mycelia. In vivo testing showed that the lesion size of muskmelons exposed to VOCs from *T*. *asperellum* T76-14 was significantly smaller than that of the control. Muskmelons exposed to VOCs from *T*. *asperellum* T76-14 showed no fruit rot after incubation at seven days compared to fruit rot in the control. This study demonstrated the ability of *T*. *asperellum* T76-14 to produce volatile antifungal compounds, showing that it can be a major mechanism involved in and responsible for the successful inhibition of *F. incarnatum* and control of postharvest fruit rot in muskmelons.

## 1. Introduction

The muskmelon (*Cucumis melo*) is a species of melon that has been bred into several cultivated varieties worldwide. In Thailand, the cultivation of muskmelons has increased due to market demand, as observed by the Department of Agricultural Extension, Ministry of Agriculture and Cooperative, Thailand. However, Thailand is located in tropical and subtropical zones, in which the weather is favorable for pathogen germination and disease dispersal. The cultivation of muskmelons is complicated by several diseases that reduce both the quality and quantity of pre-harvest and postharvest production. Postharvest fruit rot is considered one of the most destructive diseases that negatively impact saleable stock. This disease has recently been reported to be caused by *Fusarium incarnatum* [1] in Thailand.

The application of high doses of chemical fungicide has effectively controlled plant fungal diseases. However, they cause a negative effect in terms of the pesticide resistance of pathogens [2], and can have harmful side effects for humans [3]. Biological control is considered an alternative way to control postharvest fruit rot disease. It is well known that *Trichoderma* species have many helpful qualities, including antibiosis [4,5,6,7], competition for nutrients and space [8], mycoparasitism [9], and induction of systemic resistance [10,11,12] to control disease pathogens, as well as to promote plant growth ability [13].

Endophytes are well known as a source of novel biologically active secondary metabolites that confer major ecological benefits on their host plants [14]. Antibiosis is one of the strongest mechanisms of the *Trichoderma* species, and its endophytic relationship in nature provides a better combination of defense responses against several plant pathogenic fungi [11,15]. The compounds released by endophytic fungi may benefit the host plant by contributing antifungal activity [16], and offer a defense against plant pathogens [17]. Endophytes are a successful biological control agent (BCA) also capable of secreting volatile organic compounds (VOCs) and cell-wall-degrading enzymes to inhibit the fungal growth involved in antibiosis. Some *Trichoderma* species produce nonvolatile and volatile compounds responsible for inhibiting pathogens [4,5,6,16,18,19,20,21], and may directly trigger the defense system in host plants [6,11,22].

*Trichoderma* species play an important role as a BCA controlling diseases in a wide variety of plants, but the mode of action for this antagonism remains unclear. It is known that several *Trichoderma* species produce volatile organic compounds (VOCs) responsible for inhibiting pathogens, activating a defense response as well as increased plant growth. Therefore, the present study focuses on the potential of VOCs emitted by endophytic *Trichoderma* as a biofumigant against postharvest fruit rot disease in muskmelons caused by *F. incarnatum*. Endophytic fungi *Trichoderma* spp. were screened for their antifungal activities, and attempts were made to determine the antagonistic mechanisms that may be responsible for suppressing *F. incarnatum* and controlling postharvest fruit rot.

## 2. Materials and Methods

### 2.1. Trichoderma Species and Pathogen Isolates

In this study, seven *Trichoderma* species were tested for their ability to inhibit *F*. *incarnatum* [1]. Six endophytic fungal isolates of the *Trichoderma* species, *Trichoderma asperellum* T1 [11]; *T. asperellum* T76-14 (LC158827); *Trichoderma* sp. PSU-P1; *Trichoderma* sp. T76-1 (LC158829), T76-12/2 (LC158830), and V76-12 (LC158826); one soil-borne *T. harzianum* TM2/1; and the fungal pathogen *F. incarnatum*, were obtained from the Culture Collection of Pest Management, Faculty of Natural Resources, Prince of Songkla University, Thailand. The *Trichoderma* species and *F. incarnatum* were cultured on potato dextrose agar (PDA) (Himedia, Mumbai, India) for three days prior to this study.

### 2.2. In Vitro Screening of Trichoderma spp. against F. incarnatum by Dual-Culture Assay

Seven isolates of *Trichoderma* spp. were screened for their inhibitory effects on the growth of *F. incarnatum* through a dual-culture assay on PDA plates [23]. The dual-culture plates were then incubated at an ambient temperature (28 ± 2 °C) for seven days. The experiment was subjected to complete randomized design (CRD) with five replicates, and the experiment was repeated twice. Colony radii of *F. incarnatum* on the control and test plates were measured, and the percentage of inhibition was calculated by the formula previously described by Rahman et al. [24]:

(1) $$\mathrm{Percentage}\;\mathrm{inhibition}\;(\%)\;=\;\frac{R1-R2}{R1}\;\times 100,$$

where *R*1 = radial growth of *F. incarnatum* in control and *R*2 = radial growth of *F. incarnatum* with treatment.

### 2.3. Volatile Antifungal Bioassay

In order to test the volatile effects of the *Trichoderma* species on the growth of *F. incarnatum*, the sealed plate method was conducted [25], with some modifications. The *Trichoderma* species were grown on potato dextrose agar (PDA) in 9-cm Petri dishes (Citotest, Jiangsu, China) for three days. An agar plug (0.5 cm in diameter) was cut from the culture plate, inserted centrally, and the lid of each petri dish was removed. We replaced the bottom with one containing PDA inoculated with tested fungi, and the two bottom plates were then sealed together with Parafilm and incubated at 28 ± 2 °C for five days. For the control, the lids of the control plates without *Trichoderma* inoculation were replaced in the same manner. The tested plates were also incubated at ambient temperature (28 ± 2 °C) for five days. Colony diameters of *F. incarnatum* were measured and converted to the percentage of inhibition through the following formula:

(2) $$\mathrm{Percentage}\;\mathrm{inhibition}\;(\%)\;=\;\frac{Dc-Dt}{Dc}\;\times 100,$$

where *Dc* = mycelial growth of *F. incarnatum* on control plate, and *Dt* = mycelial growth of *F. incarnatum* on the test plate. Each treatment was performed in five replicates, and the results are presented as the mean ± standard error (*n* = 5).

### 2.4. GC/MS Analysis

In order to examine the volatile and semi-volatile antifungal compounds within the *Trichoderma* species, gas chromatography spectrophotometry (GC/MS) was conducted. The *Trichoderma* species was cultured in a 20-mL chromatography vial, 20 mm in diameter (PerkinElmer, Waltham, MA, USA), and incubated at ambient temperature (28 ± 2 °C) for 14 days according to the method of Wonglom et al. [6] Solid-phase microextraction (SPME) was performed to extract the volatile organic compounds (VOCs), as previously described by Arthur et al. [6,26] SPME fiber (DVB/CAR/PDMS fiber) was exposed to the vapors above each *Trichoderma* culture for 30 min, and inserted into the injection port of the gas chromatograph SQ8 (PerkinElmer, Waltham, MA, USA), equipped with a DB-Wax capillary column (30 m × 0.25 mm i.d., 0.25 μm film thickness). The oven temperature was initially maintained at 40 °C, then increased to 230 °C, at a rate of 7 °C min^−1^. The injector temperature was 230 °C. The carrier gas was ultra-high-purity helium with an initial column head pressure of 60 kPa, at a flow rate of 1 mL min^−1^. Electron impact (EI) mass spectra were collected at 70 eV ionization voltage over the *m*/*z* range 29–550. The ion source and quadrupole temperatures were both set to 200 °C. Volatiles and semivolatiles were identified based on the computer searches dictated by The National Institute of Standards and Technology (NIST) Mass Spectral Library Search Chromatogram.

### 2.5. Sealed Plate Method of Commercial Volatile against Mycelial Growth of F. incarnatum

To test the effect of dominant volatile compound participated in antifungal activity against *F. incarnatum*, the sealed plate method was conducted as shown in Section 2.3. The compound phenylethyl alcohol (PEA) was purchased from Sigma-Aldrich (St. Louis, MO, USA). The effect of commercial PEA versus the other volatile antifungal compounds 2-ethylhexanol, 1-nonanol, 6-PP, and 2-methyl-1-butanol (Sigma-Aldrich, St. Louis, MO, USA) [6] was tested through the sealed plate method. PEA was dissolved in 95% ethanol and we adjusted the dilution to 10^−1^, 10^−2^, and 10^−3^. Each volatile compound was applied on a sterile cotton pad (20 µL) and subjected to the method of Wonglom et al. [6] Application of 95% ethanol served as a negative control. The tested plates were then incubated at 28 ± 2 °C for seven days. Each treatment was composed of five replicates and the experiment was repeated twice. Colony diameters of *F. incarnatum* were measured and the percentage inhibition was calculated as described in Section 2.2.

### 2.6. In Vivo Test of Volatile against Postharvest Fruit Rot

**Figure 1:**
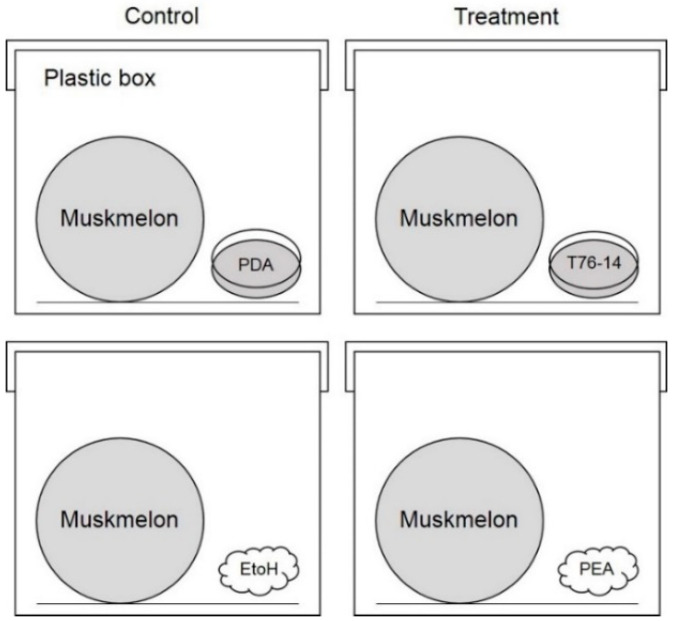
Overview of experimental setup and VOC effect against *Fusarium incarnatum* on muskmelons. Schematic overview of the plate-within-a-box system.

To test the effect of VOCs and commercial volatiles against postharvest fruit rot in muskmelon fruits, an in vivo test was conducted. *F. incarnatum* was cultured on PDA and incubated at ambient temperature (28 ± 2 °C) for seven days. Conidia of *F. incarnatum* were harvested and we adjusted the concentration to 1 × 10^6^ conidia mL^−1^. A conidia suspension (20 μL) was applied to muskmelon fruit, and each experiment, consisting of three fruits (three replicates), was repeated three times. Inoculated muskmelon fruits were incubated with VOCs from *Trichoderma* (eight petri dishes per treatment) or commercial volatile at 10^−1^ dilution in 50 μL applied on a cotton pad (four cotton pads) in a 24 × 35 × 18 cm plastic box (Figure 1) to share the atmosphere for three days. Inoculated muskmelon fruits incubated with PDA alone or 95% ethanol alone served as controls. Lesion development was measured and compared between the treatment and the control.

### 2.7. Morphological Study of F. incarnatum Exposed to Volatiles

To test the effect of VOCs or commercial volatiles on the morphology changes of *F. incarnatum* mycelia, the sealed plate method was conducted as described in Section 2.3. Changes in morphology between VOCs and commercial volatiles treated and untreated with mycelia from this study were observed by a Leica DM750 compound microscope (Leica Microsystems, Wetzlar, Germany).

### 2.8. Effect of VOCs Emitted from T. asperellum T76-14 on Fruit Rot

To test the effect of VOCs emitted from *T. asperellum* T76-14 on fruit rot in muskmelons, five muskmelon fruits were disinfected with 70% ethanol prior to VOC exposure. Muskmelon fruits were incubated with VOCs from *Trichoderma* or commercial volatiles, as described in Section 2.6, in a 24 × 35 × 18 cm plastic box to share the atmosphere for seven days. Fruit rot was observed from the day after treatment and recorded.

### 2.9. Statistical Analysis

Data including fungal growth, percentage inhibition, and lesion size were subjected to one-way analysis of variance (ANOVA). Statistically significant differences between treated samples and the untreated control were analyzed by Tukey’s test with a threshold of *p* < 0.05 [27].

## 3. Results

### 3.1. Trichoderma Species Inhibit Mycelial Growth of F. incarnatum

**Figure 2:**
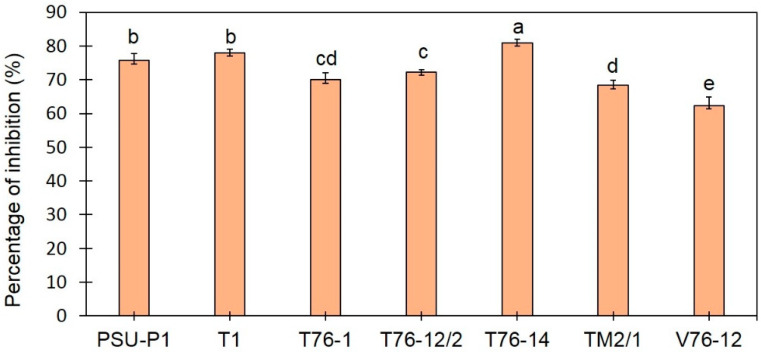
Percentage inhibition of *Trichoderma* spp. against *Fusarium incarnatum* in PDA by dual-assay plates. Different letters indicate statistically significant differences among treatments (*p* < 0.05) using Tukey’s test.

**Figure 3:**
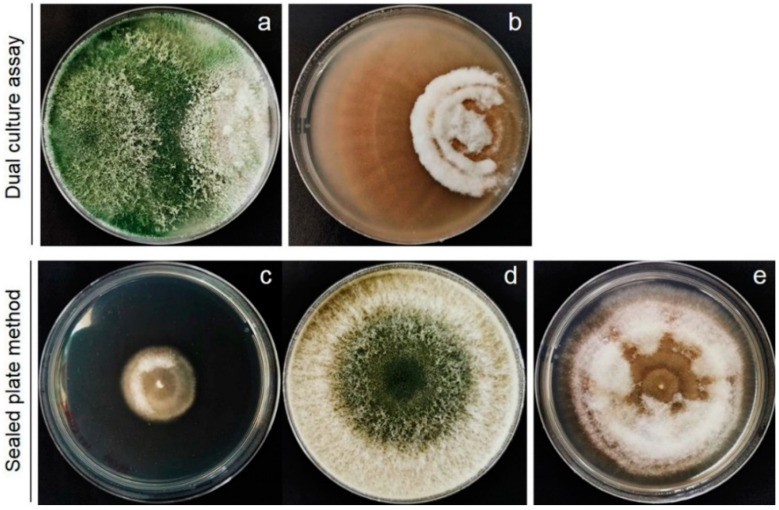
In vitro test antagonistic *Trichoderma* against *Fusarium incarnatum*, *Trichoderma asperellum* T76-14, and *F. incarnatum* in dual culture plate (**a**), *F. incarnatum* in PDA plate alone (**b**), *F. incarnatum* (**c**) and *T. asperellum* T76-14 (**d**) in sealed plate method and *F. incarnatum* in PDA plate alone (**e**).

Based on the primary screening for BCA by dual-culture assay, *Trichoderma* spp. inhibited the fungal growth of *F. incarnatum* on the PDA plates. The percentage of inhibition ranged from 62% to 81%. Among the seven tested isolates, *T. asperellum* T76-14 effectively suppressed the growth of *F. incarnatum* by 81% (Figure 2 and Figure 3), statistically higher than those of the other isolates (*p* < 0.05), and was therefore selected for further analysis.

### 3.2. Volatiles Emitted by T. asperellum T76-14 Inhibit Mycelial Growth of F. incarnatum

To test the effects of the VOCs emitted by *T. asperellum* T76-14 on the fungal growth of *F. incarnatum*, the sealed plate method was conducted. After five days of incubation in ambient temperature, the colony diameters of *F. incarnatum* in the tested plates were smaller than those of the control bioassay plates (Figure 3). In the presence of VOCs emitted by *T. asperellum* T76-14, the growth of *F. incarnatum* was slower than without VOCs on PDA (Figure 3). The colony diameters of *F. incarnatum* in the control and test plates were measured and converted to represent the percentage of inhibition of fungal growth. The results demonstrated suppressed mycelial growth of *F. incarnatum*, with the greatest percentage of inhibition being 62.5%.

### 3.3. Identifying Volatile Organic Compounds

**Figure 4:**
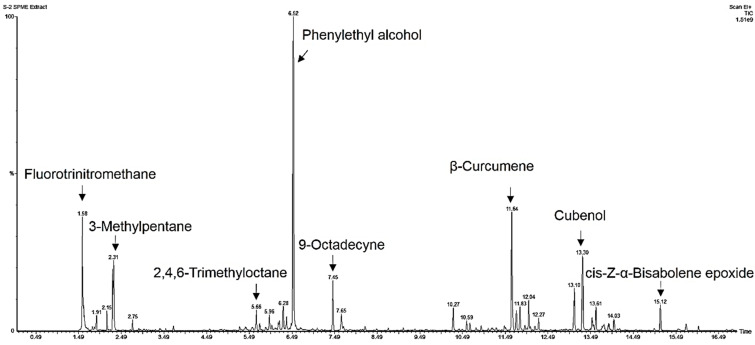
Total ion chromatogram of volatile compounds identified from *T. asperellum* T76-14.

**Figure 5:**
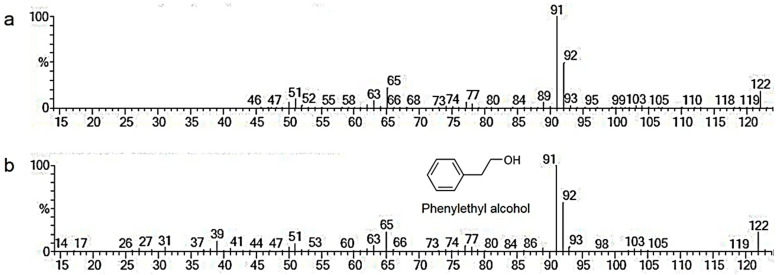
Mass spectrum for the compound at 6.52 min (**a**) and phenylethyl alcohol (PEA) with its structure (**b**).

The results above demonstrate that the VOCs emitted by *T. asperellum* T76-14 inhibited the fungal growth of *F. incarnatum*, which suggests the antifungal activity of the volatile organic compounds. To characterize the VOCs of *T. asperellum* T76-14, SPME was performed to collect the VOCs, and they were analyzed by GC/MS. The results shown in Table 1 and Figure 4 and Figure 5 are from the preliminary screening of the VOCs produced by *T. asperellum* T76-14 that were responsible for inhibiting *F. incarnatum*. The chromatogram is presented in Figure 4. A total of 17 compounds with >70% were tentatively identified using the NIST library (Table 1). The compounds detected in *T. asperellum* T76-14 are members of the compound classes alcohols, alkane, and terpene, as illustrated in Table 1. The most dominant volatile compounds detected in this study were phenylethyl alcohol (PEA) at 6.52 min with 23.188% peak area, followed by fluorotrinitromethane, at 1.578 min with 8.778% peak area, and β-Curcumene, a sesquiterpene, at 11.641 min with 8.488% peak area. Figure 5 shows the mass spectrum of some of the major compounds, and the structure of the most dominant compound, PEA.

### 3.4. Effect of Commercial Volatile Compounds on Antifungal Activity against F. incarnatum

Commercial volatile compounds that have been reported as antifungal compounds (2-ethylhexanol, 1-nonanol, 6-PP, and 2-methyl-1-butanol) and PEA were tested against *F. incarnatum* growth in vitro on PDA plates. The results showed that all commercial volatile compounds inhibited the mycelial growth of *F. incarnatum* (Table 2). High percentage inhibition of volatile compounds depended on high concentrations. A dilution of 10^−1^ of all volatile compounds strongly inhibited *F. incarnatum*, with the percentage inhibition ranging from 21.68% to 74.29%. 2-ethylhexanol was found to be the best volatile compound for inhibiting *F. incarnatum*, with a percentage inhibition of 74.28%. The PEA showed a percentage inhibition of *F. incarnatum* of 56% at a dilution of 10^−1^, equivalent to a concentration of 1.5 mg mL^−1^. This confirmed that PEA is a volatile antifungal compound against *F. incarnatum*.

**Figure 6:**
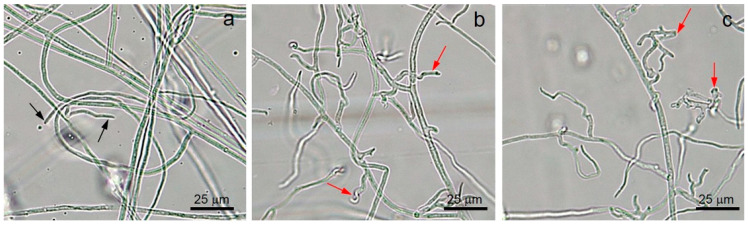
Morphological observation of *Fusarium incarnatum* mycelia, in control (**a**), exposed to VOCs emitted by *T. asperellum* T76-14 (**b**), and PEA (**c**), black arrows indicate normal hyphal tips and red arrows indicate abnormal hyphal tips.

Fungal mycelia among the VOCs released from *T. asperellum* T76-14, PEA, and the control was observed by a compound microscope. The fungal mycelia of *F. incarnatum* exposed to VOCs and PEA showed an abnormal shape, whereas the control showed no change of mycelia and remained a normal shape (Figure 6).

### 3.5. VOCs of T. asperellum T76-14 Reduced Postharvest Fruit Rot in Muskmelon Fruits

**Figure 7:**
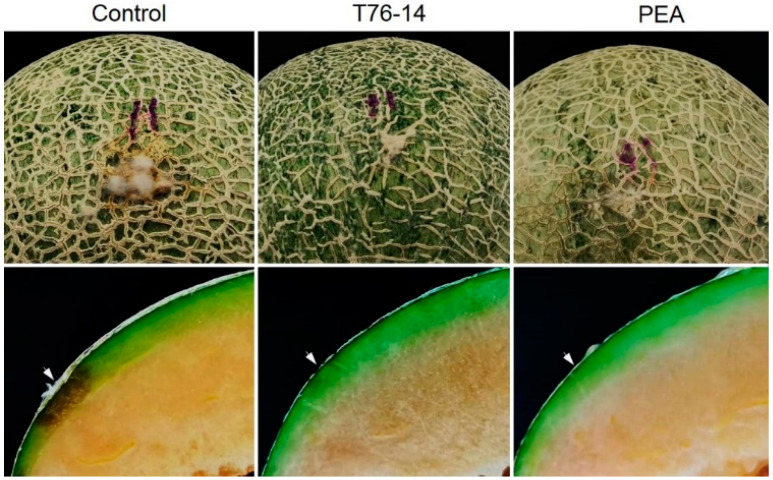
Lesion development after inoculation with *F. incarnatum*, control without VOCs and PEA, muskmelons exposed to VOCs of *Trichoderma asperellum* T76-14 and phenylethyl alcohol (PEA). Arrows indicate the inoculation points.

**Figure 8:**
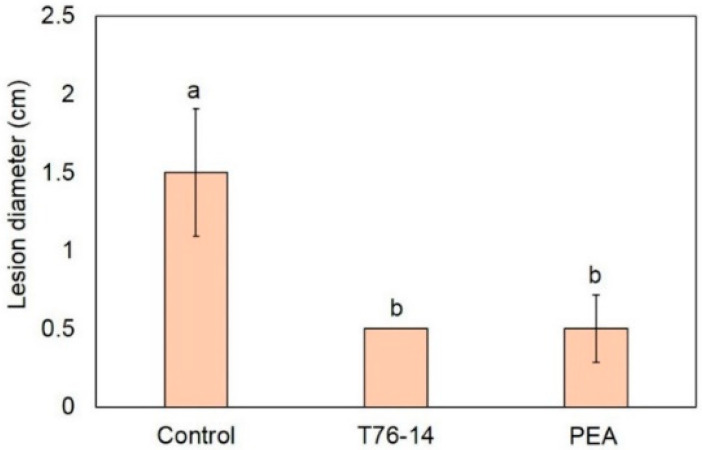
Lesion diameter on muskmelons in the control versus muskmelons exposed to *Trichoderma asperellum* T76-14 (T76-14) and phenylethyl alcohol (PEA). Different letters indicate statistically significant differences among treatments (*p* < 0.05) using Tukey’s test.

As seen above, the VOCs emitted from *T. asperellum* T76-14 and PEA showed antifungal activity against *F. incarnatum*, the pathogen of postharvest fruit rot in muskmelons. Therefore, they were selected to test in vivo against fruit rot in muskmelons by observing the lesion size after inoculation with a spore suspension of *F. incarnatum*. After incubation for three days, the lesions of VOCs- and PEA-treated muskmelons were statistically significantly smaller than in the control (Figure 7 and Figure 8). The lesion sizes of the control, VOCs-treated, and PEA-treated muskmelons were 1.5, 0.5, and 0.5 cm, respectively (Figure 8).

### 3.6. VOCs of T. asperellum T76-14 Suppressed Fruit Rot in Muskmelons

Based on the results above, we hypothesized that the VOCs emitted from *T. asperellum* may preserve muskmelons from fruit rot during postharvest storage. In order to test the effect of VOCs on muskmelons during postharvest, fruit rot was observed from the day after exposure with VOCs or PEA. The results showed that, after incubation at an ambient temperature for seven days, rotten tissue was observed in control muskmelons and muskmelons exposed to PEA, whereas VOC-treated muskmelons did not have rot (Figure 7). Fungal mycelia-covered tissues at peduncle were most prominent in the control, followed by PEA-treated muskmelons, causing tissue rot inside the muskmelon. Muskmelons exposed to VOCs from *T. asperellum* T76-14 showed less fungal mycelia on the fruit peduncle and no rotten tissue inside seven days after incubation.

## 4. Discussion

In the present study, *T. asperellum* T16-14 exhibited antifungal activity against the mycelial growth of *F. incarnatum*, the causal microorganism of postharvest fruit rot in muskmelons. *T. asperellum* T16-14 effectively inhibited mycelial growth of *F. incarnatum* in vitro by a dual-culture assay, which suggested a competition mechanism (Figure 2). The sealed plate method also revealed the antibiosis mechanism of *T. asperellum* T16-14 against *F. incarnatum* (Figure 3). The dominant volatile compound released by *T. asperellum* T16-14, tentatively identified as PEA based on SPME GC/MS, was involved in antifungal activity against *F. incarnatum* (Figure 4 and Figure 5). VOCs released by *T. asperellum* T16-14 caused abnormal changes in the *F. incarnatum* mycelia (Figure 6), reducing the postharvest fruit rot in muskmelons (Figure 7 and Figure 8).

In a primary screening by dual-culture assay, *T. asperellum* T76-14 grew faster than *F. incarnatum* on PDA (Figure 3), suggesting a competition mechanism. This mechanism is common in several species of *Trichoderma* against plant pathogens [4,5,28]. In the study above, *T. asperellum* T76-14 was found to produce VOCs (Figure 3 and Table 1) that slow the fungal growth of *F. incarnatum*. This production involved an antibiosis mechanism within the *Trichoderma* species. It has been established that several *Trichoderma* species emit volatile antifungal compounds that inhibit pathogen growth [4,5,6,25]. In this study, volatiles emitted from *T. asperellum* T76-14 inhibited the fungal growth of *F. incarnatum* by 62.5% (Figure 2), as determined through the sealed plate method. The VOCs emitted by *T. asperellum* T76-14 slow the fungal growth of *F. incarnatum*, similar to PEA. Both VOCs and PEA caused abnormal changes in the fungal mycelia (Figure 6), which may result in inhibiting the fungal growth of *F. incarnatum*. As shown in Figure 4 and Table 1, GC/MS revealed some minor peaks, which may have contributed to antifungal activity due to a low percent peak area. This revealed that the VOCs emitted by *T. asperellum* T76-14 were responsible for the antifungal activity against *F. incarnatum*. The GC/MS results presented 17 compounds; however, according to the literature, the only antifungal compound found in this study was PEA (Figure 4 and Figure 5).

PEA, also known as an aromatic alcohol, is an organic compound found as a colorless liquid with a rose-like odor. This compound occurs widely in nature as an essential oil from several plant species such as roses, jasmine, carnations, and hyacinths [29]. PEA has been used to modify certain flavor compositions of foods with low toxicity [30,31]. It has been shown that PEA has antimicrobial properties [29,30,32,33,34]. An in vitro study revealed that PEA inhibits fungal growth and the synthesis of RNA, DNA, and the protein of *Neurospora crassa* [32]. PEA has been reported to show strong antifungal activity against *Ganoderma boninense* [35], significantly reducing fungal growth to prolong the postharvest life of strawberries [34], and controlling the blue mold decay caused by *Penicillium digitatum* and *P. italicum* in citrus fruits [36]. Our result is in agreement with previous reports that PEA inhibited the fungal growth of *F. incarnatum* in vitro (Table 2) and reduced lesions on muskmelon fruits (Figure 7 and Figure 8). This also proved that PEA in VOCs released from *T. asperellum* T76-14 had antifungal activity against *F. incarnatum*.

**Figure 9:**
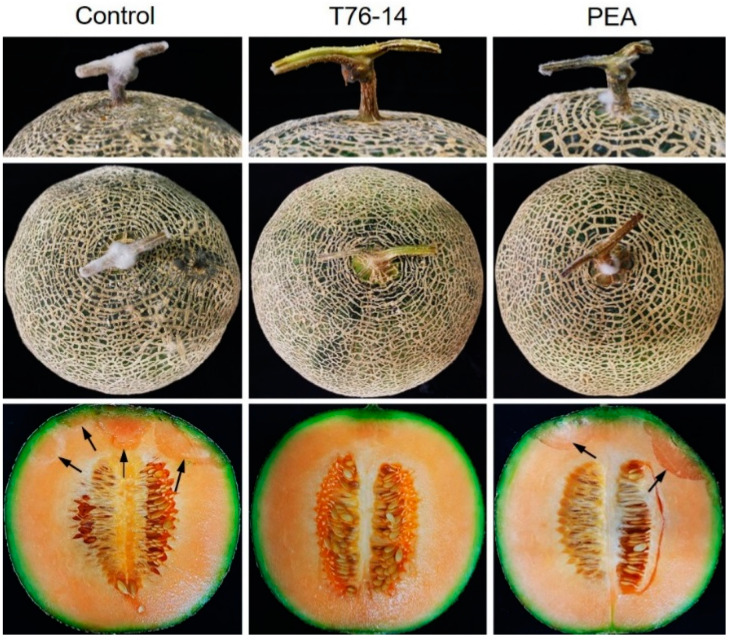
Effect of VOCs and PEA on postharvest fruit rot in muskmelons, T76-14 = *Trichoderma asperellum* T76-14 and PEA = phenylethyl alcohol. Arrows indicate the rotten tissue.

PEA has been reported to be produced by several *Trichoderma* species. For instance, PEA emitted by *Trichoderma* sp. [29], *T. atroviride* [37], *T. harzianum* [38], *T. spirale* [5], and *T. virens* [36] displayed antifungal ability against several plant pathogens. In this study, PEA emitted by *T. asperellum* T76-14, is a dominant compound with 23.188% peak area (Table 2), and has been reported to have antifungal activities [34,35]. Therefore, the ability of *T. asperellum* T76-14 to produce volatile antifungal compounds would further promote antifungal activity against *F. incarnatum*. In this study, we investigated the effect of VOCs emitted by *T. asperellum* T76-14 on fungal growth, morphology changes of fungal mycelia, and the reduction in postharvest fruit rot, as well as the prolonged postharvest life of muskmelons. In comparison with commercial PEA, VOCs of *T. asperellum* T76-14 prevented fruit rot in muskmelons, which may be due to a complex mix of VOCs with other volatiles, working synergistically to inhibit fungal growth. Commercial volatile PEA can limit lesion development at three days after treatment (Figure 7); however, at seven days it showed fruit rot similar to the control (Figure 9). This may result from PEA vapor after incubation in a long period in a shared-atmosphere plastic box. The use of fresh *Trichoderma* may produce VOCs continuously; however PEA was applied once so the concentration may reduce after a long period of incubation.

Several reports have clarified the effect of microbial volatiles as a biofumigant controlling postharvest diseases and prolonging postharvest life. For instance, Li et al. [39] used VOCs produced by *Ceratocystis fimbriata* as biofumigation on postharvest disease of fruit. The authors showed that VOCs inhibited the growth of *Monilinia fructicola* and *Penicilium digitatum* (causing peach brown rot and citrus green mold, respectively) [39]. Volatile compounds produced by *Aureobasidium pullulans* increased fruit waxes’ complexity, reducing pathogen attacks on stone fruit [40]. Furthermore, VOCs emitted from *T. atroviride* inhibited the mycelial growth of *Phytophthora infestans* in postharvest potato tubers [41]. The authors investigated VOCs by GC/MS and found abundant compounds, namely 3-mrthyl-1-butanol, p-6-pentyl-2-pyrone, 2-mthyl-1-propanol, and acetone, which were responsible for serious morphological and ultrastructural damage of the pathogens [41]. Our findings are in agreement with [41]: *T. asperellum* T76-14 produced VOCs, which contained dominant volatiles of PEA. VOCs of *T. asperellum* T76-14 are responsible for inhibiting postharvest fruit rot pathogens and could prolong the postharvest life of muskmelons. During postharvest storage, some fungi or mold can infest the fruit surface and penetrate into tissues, causing fruit rot. This finding indicates that VOCs released from *T. asperellum* T76-14 contain PEA to clean the fruit surface and reduce fungal infection, which may prolong the postharvest life of muskmelons.

## 5. Conclusions

Herein, we described the ability of VOCs emitted by *T. asperellum* T76-14 involved in antifungal activity against the postharvest fruit rot pathogen (*F. incarnatum*) of muskmelons to reduce fruit rot during postharvest storage by inhibiting postharvest pathogens. However, the extraction of valuable compounds, and the quality and quantity of volatiles emitted by this strain, have yet to be investigated. In order to apply VOCs from *T. asperellum* T76-14 as a biofumigant to control postharvest disease and maintain postharvest quality on a large scale, further experiments need to be conducted.

## Figures and Tables

**Table 1:** Volatile compounds produced by *Trichoderma asperellum* T76-14 tentatively identified through SPME GC/MS analysis.

RT (min)	Volatile Compounds	Formula	% Math	% Area
1.58	Fluoro(trinitro)methane *	CFN_3_O_6_	95	8.78
2.29	Pentan-1-ol	C_5_H_12_O	86.4	2.98
2.31	3-Methylpentane	C_6_H_14_	89.2	4.10
5.66	2,4,6-Trimethyloctane	C_11_H_24_	83.7	1.34
6.28	5,7-Dimethylundecane	C_13_H_28_	84.4	1.60
6.52	Phenylethyl alcohol	C_8_H_10_O	94.8	23.19
7.45	Octadec-9-yne	C_18_H_34_	74.9	3.09
7.65	1-Methylideneindene	C_10_H_10_	92.8	1.34
10.27	3-Isopropyl-6,8a-dimethyl-1,2,4,5,8,8a-hexahydroazulene	C_15_H_24_	85.8	1.41
11.64	β-Curcumene	C_15_H_24_	84.4	8.49
11.83	α-Bisabolene	C_15_H_24_	83.8	1.69
12.04	Sesquisabinene B	C_15_H_24_	83.5	1.74
13.10	Zingiberenol	C_15_H_26_O	81.8	3.11
13.30	Cubenol	C_15_H_26_O	83.1	6.04
13.61	Undeca-3,4-diene-2,10-dione, 5,6,6-trimethyl	C_14_H_22_O_2_	75.1	1.44
14.03	Allyldimethyl(prop-1-ynyl)silane	C_8_H_14_Si	71.4	0.77
15.12	cis-Z-α-Bisabolene epoxide	C_15_H_24_O	71.3	1.73

* The result of three replicates.

**Table 2:** Comparison of phenylethyl alcohol (PEA) and other commercial volatiles against *Fusarium incarnatum*.

Commercial Volatiles	Percentage Inhibition (%) ^a^		
	10^−1^	10^−2^	10^−3^
1-Nonanol	55.42 ± 13.69 ^b^	29.71 ± 3.32 ^b^	19.67 ± 5.69 ^c^
2-Ethylhexanol	74.29 ± 11.12 ^a^	29.71 ± 5.01 ^b^	24.89 ± 9.04 ^a^
2-Methyl-1-butanol	39.12 ± 8.43 ^c^	20.88 ± 4.86 ^d^	16.86 ± 1.20 ^d^
6-Pentyl-2H-pyran-2-one	21.68 ± 5.25 ^d^	32.53 ± 14.65 ^a^	20.08 ± 6.18 ^b^
Phenylethyl alcohol	56.00 ± 5.25 ^b^	24.00 ± 6.92 ^c^	12.00 ± 4.00 ^e^

^a^ Percentage inhibition of mycelial growth was calculated using 95% ethyl alcohol as a negative control. Means within the same column followed by the same superscript letters are not significantly different at *p* < 0.05 using Tukey’s test.

## Data Availability

Not applicable.
